# Space Radiation Protection Countermeasures in Microgravity and Planetary Exploration

**DOI:** 10.3390/life11080829

**Published:** 2021-08-14

**Authors:** Carlos A. Montesinos, Radina Khalid, Octav Cristea, Joel S. Greenberger, Michael W. Epperly, Jennifer A. Lemon, Douglas R. Boreham, Dmitri Popov, Gitika Gorthi, Nandita Ramkumar, Jeffrey A. Jones

**Affiliations:** 1 Nugevity Health Sciences, Houston, TX 77450, USA; cam@nugevity.com; 2 School of Engineering, Rice University, Houston, TX 77005, USA; radinaanidar@gmail.com; 3 Department of Surgery, Emory University School of Medicine, Atlanta, GA 30322, USA; octav.cristea@emoryhealthcare.org; 4 Department of Radiation Oncology, University of Pittsburg Medical Center, Pittsburgh, PA 15213, USA; greenbergerjs@upmc.edu (J.S.G.); eppemw@upmc.edu (M.W.E.); 5 Medical Sciences Division, Northern Ontario School of Medicine, Sudbury, ON P3E 2C6, Canada; jlemon@nosm.ca (J.A.L.); d.boreham@nosm.ca (D.R.B.); 6 Advanced Medical Technologies and Systems Inc., Richmond Hill, ON L4B 1N1, Canada; biosmedimmune@gmail.com; 7 Ignited Thinkers, Fairfax, VA 22030, USA; gitikagorthi@gmail.com; 8 Center for Space Medicine, Baylor College of Medicine, Houston, TX 77030, USA; nandita.ramkumar@bcm.edu; 9 Center for Space Medicine, Department of Urology, Baylor College of Medicine, Houston, TX 77030, USA

**Keywords:** space radiation, bioeffects, chromosomal aberrations, radioprotection, countermeasures, Lunar/Mars exploration, solar particle event, immunology, nutrition

## Abstract

Background: Space radiation is one of the principal environmental factors limiting the human tolerance for space travel, and therefore a primary risk in need of mitigation strategies to enable crewed exploration of the solar system. Methods: We summarize the current state of knowledge regarding potential means to reduce the biological effects of space radiation. New countermeasure strategies for exploration-class missions are proposed, based on recent advances in nutrition, pharmacologic, and immune science. Results: Radiation protection can be categorized into (1) exposure-limiting: shielding and mission duration; (2) countermeasures: radioprotectors, radiomodulators, radiomitigators, and immune-modulation, and; (3) treatment and supportive care for the effects of radiation. Vehicle and mission design can augment the overall exposure. Testing in terrestrial laboratories and earth-based exposure facilities, as well as on the International Space Station (ISS), has demonstrated that dietary and pharmacologic countermeasures can be safe and effective. Immune system modulators are less robustly tested but show promise. Therapies for radiation prodromal syndrome may include pharmacologic agents; and autologous marrow for acute radiation syndrome (ARS). Conclusions: Current radiation protection technology is not yet optimized, but nevertheless offers substantial protection to crews based on Lunar or Mars design reference missions. With additional research and human testing, the space radiation risk can be further mitigated to allow for long-duration exploration of the solar system.

## 1. Introduction

### 1.1. The Space Radiation Environment

Space exploration presents a unique challenge for the human body, requiring it to operate in an environment markedly different from the terrestrial one in which it evolved. On Earth, humans are exposed to relatively low levels of ionizing radiation due to the protective effects of the Earth’s atmosphere and geomagnetosphere [1]. The majority of radiation experienced by life on the terrestrial surface is in the form of electromagnetic radiation (i.e., high-energy photons without mass or charge) [2]. Beyond the Earth’s geomagnetosphere, however, the space radiation environment is not only unmitigated but also characterized by a different type of radiation in addition to the electromagnetic—namely, particle radiation (i.e., high energy particles with mass and with or without charge) [3]. In space, this particle radiation takes two principal forms: a) moderate-to-high energy protons released from the sun during solar particle events (SPEs), and b) high energy protons and heavier ions traveling through space at relativistic speeds, known as galactic cosmic rays (GCRs). Whereas SPEs occur sporadically and unpredictably, GCRs are uniformly present, having originated from distant supernova events [4]. So-called HZE particles—named as they are for their high atomic number (Z) and energy (E)—represent the subset of GCRs that consist of atomic nuclei heavier than helium. While representing only a small fraction of GCRs overall, these particles contribute significantly to the energy imparted to biological tissues and, due to their mass and charge, are both uniquely harmful and difficult to shield against, as we will see in greater detail below [5,6]. In addition to the above, the space radiation environment around the Earth is further characterized by radiation trapped in the geomagnetosphere itself, which consists primarily of high-energy protons and electrons [7]. Taken together, these sources of radiation characterize an environment that is quite hostile to human life. The ubiquity of space radiation, the significant challenges of shielding against it, and the detrimental effects it has upon biological systems combine to create one of the most important barriers to long-term human exploration of the solar system beyond low earth orbit (LEO) [8,9].

### 1.2. Measuring the Biological Effects of Radiation

The biological effects of radiation are numerous and depend upon many factors, including the radiation dose, type, amount of tissue exposed, and the susceptibility of a given organ or tissue type to radiation-induced damage. At the cellular level, radiation can cause indirect damage via the production of reactive oxygen species that overwhelm the cell’s protective mechanisms, as well as direct damage to key cellular structures, such as DNA. These injuries can lead to mutations, chromosomal aberrations, functional abnormalities, senescence, and cell death [10,11]. Clinically, radiation exposure can result in a spectrum of acute radiation syndromes with symptoms ranging from nausea, vomiting, and bleeding to immune system collapse, mental status changes, and even death [12]. Long-term effects of radiation exposure include cataract formation, an increased risk of carcinogenesis, and degenerative changes of the cardiovascular and central nervous systems, among others [11,13,14,15,16]. To date, the majority of our knowledge regarding the clinical effects of radiation exposure is derived from terrestrial exposures to electromagnetic radiation. These include studies of atomic bomb survivors, industrial exposures or accidents, and radiation used for medical therapy [2,17]. Emerging evidence, however, suggests that particle radiation may be more biologically detrimental than its electromagnetic counterpart, even at equivalent energies, complicating our ability to extrapolate from the aforementioned knowledge base to the space radiation environment that will affect the crews of long-duration missions [18,19].

The absorbed dose of ionizing radiation is measured in gray (Gy), which is defined as 1 joule (J) of energy absorbed per kilogram of matter. Conventionally, the Gy is used to express any directly measured dose or any dose threshold associated with deterministic or acute biological effects, where the doses received are high (e.g., lethal total body dose range > 2 Gy). Absorbed dose alone, however, does not account for differences between radiation types or tissues and is therefore insufficient for predicting the long-term biological effects of lower radiation doses, particularly those that are probabilistic in nature, such as the risk of carcinogenesis. In order to account for this, quality/weighting factors are applied to the absorbed dose to adjust for different radiation types (QF/WR) as well as variations in tissue susceptibility (WT), yielding what is known as the equivalent and effective dose, respectively [20,21]. Both the equivalent and effective doses are expressed in sievert (Sv), which, like the Gy, is measured in J/kg. Unlike the Gy, however, the Sv is not a directly measured dose but rather a calculated dose used to estimate the long-term risks associated with radiation exposure where exposure of 1 Sv is approximately equivalent to a 5% risk of developing a fatal malignancy [20]. To help set appropriate limits on radiation exposure, the principle of “as low as reasonably achievable” (ALARA) is applied in various occupational settings. Terrestrial radiation workers are limited to an exposure of 50 mSv/year, whereas the annual exposure limit for US astronauts is 500 mSv, with career limits set such that there is a maximum of 3% increased lifetime risk of exposure-induced death from cancer [3]. Depending on astronaut age and sex, this upper limit can therefore range from 600–1200 mSv [22]. European, Russian, and Canadian astronaut career exposure limits, by contrast, are uniformly set at 1 Sv [23].

### 1.3. Exploration Class Mission Profiles and the Need for Countermeasures

Since the last Apollo mission in 1972, human spaceflight activities have been confined to LEO, and have varied in duration depending on the mission profile, ranging from 1–2 weeks for most Space Shuttle missions to 3–12 months for most expeditions on the International Space Station (ISS), with a number of long-duration stays on the space station Mir. Despite the increased radiation environment, however, astronauts in LEO are still generally protected by the Earth’s geomagetosphere, with the majority of the radiation dose absorbed during a spacecraft’s brief passage through the South Atlantic Anomaly—a region where trapped radiation in the Earth’s geomagnetic field dips to lower altitudes [24]. Crew members on an average Space Shuttle mission incurred a skin dose of 4.3 mSv, and current crews on the ISS incur an average skin dose of 0.5–1 mSv/day, varying with baseline solar activity [3]. Beyond LEO, the highest skin dose incurred during the Apollo 14 mission, which lasted just over 9 d and included 9 h of extra-vehicular lunar surface operations, was 14 mSv [3]. More recent data from the Lunar Lander Neutrons and Dosimetry experiment aboard China’s Chang’E 4 Lander measured a dose equivalent of 1.4 mSv/day on the lunar surface, approximately double the daily dose equivalent of 0.7 mSv/day measured on the ISS during the same period of solar minimum, with the relative contribution from GCRs increased by a factor of 2.6 [25]. The overall dose incurred during a long-duration lunar mission would vary based on mission duration, shielding strategies, surface conditions, and the occurrence of one or more SPEs during the mission. The timing of the mission with respect to the solar cycle is also of notable importance, as the solar maximum is associated with a decrease in the flux of GCRs but an increased risk of exposure to SPEs, and vice versa. One scenario envisions a 12-month mission on the lunar surface in a habitat covered by regolith, with a heavily shielded (10 gm/cm^2^ of aluminum or equivalent) “storm shelter” sleep station in which crews would spend approximately one-third of their time. Total mission exposure is estimated at 420 mSv to the skin, however, this rises to a total of 1.57 Sv in the presence of a single SPE event analogous to that of August 1972, which occurred between the missions of Apollo 16 and 17 [3]. Similarly, risk estimates associated with a round-trip Mars mission depend on several assumptions regarding transit time, trajectory, surface activity, and shielding strategies. The Radiation Assessment Detector (RAD) aboard the Mars Science Laboratory’s Curiosity rover has provided detailed measurements of the radiation environment in transit to and on the Martian surface under conditions of low-to-moderate solar activity and complex shielding [26,27]. Based on current propulsion technology and existing Mars mission architectures, a long-duration surface stay mission, characterized by 500 days on the surface and 180 days of transit in each direction, would result in a total dose equivalent of 1.01 Sv [27,28]. While significant uncertainty remains, both in terms of the dose received and its biological effects, it is clear that long-duration missions beyond LEO will likely expose crews to greater doses of radiation than those experienced by current astronauts engaged in LEO activities, and that many of these missions have the potential to meet or exceed the current radiation limits set for career astronauts. As our ventures beyond LEO become more frequent and more prolonged, strategies to mitigate the biological consequences of radiation will become central not just to long-term astronaut health but also to the success of any mission.

Fundamentally, two strategies characterize all radiation protection efforts: (a) reducing exposure or (b) mitigating its effects. In space, the former predominantly takes the form of shielding and/or minimizing the amount of time spent in interplanetary space, where exposure is greatest [29,30]. The risk from exposure to SPEs can also be affected by augmenting the distance to the radiation source (i.e., the Sun), with certain Mars mission trajectories involving a perihelion passage inside the orbit of Venus, in exchange for less mission time overall [28]. Distance is not a modifiable factor with respect to GCRs, however, since these are ubiquitous. Mitigating the effects of radiation can take the form of preventive strategies, such as nutritional support or prophylactic medications that boost the body’s ability to respond to radiation, or reactive strategies where medications are given to treat or support the body’s response after a radiation insult has been incurred [31,32,33]. In this review, we will provide an overview of the existing array of these strategies, discuss the special considerations that apply in the space radiation environment, and highlight new developments in radiation protection as well as promising avenues for future research.

## 2. Countermeasures

### 2.1. Shielding

Passive shielding involves the placement of physical material between a subject and a radiation source, Terrestrial radiation workers or recipients of radiation therapy can be effectively shielded using simple barrier materials, such as lead, with shield effectiveness increased in proportion to shielding material thickness [34]. Unfortunately, this relationship is more complex in the space radiation environment due to the properties of GCRs. As a consequence of their high energy and mass, GCRs interact with shielding material to produce showers of secondary radiation particles, including neutrons, which, while lower in energy, can increase the effective dose by transforming one incident beam into many [35,36,37]. As such, shielding against GCRs is only partially effective, and effectiveness drops off with increasing shield thickness [30,35]. This effect can be partially mitigated when the shielding material is of low atomic number (e.g., hydrogen) [38,39]. However, as with all solutions involving spacecraft design, trade-offs must be considered in light of mission needs, technical feasibility, and mass considerations.

The ideal shielding material can be conceived as having a high energy absorption capacity while being light, flexible, of low atomic number, stable, non-reactive, and resistant to degradation over time. The standard spacecraft construction material is aluminum, and the walls of the typical spacecraft, therefore, provide approximately 5 g/cm*2* of aluminum shielding, although some areas of the ISS are effectively shielded with up to 20 g/cm^2^ due to the presence of other modules and payloads [30]. On the ISS, the crew sleeping quarters are additionally lined with polyethylene—a hydrogen-rich material—which confers a radiation dose reduction of approximately 20% [29]. Water represents an alternative hydrogen-rich material that can be employed for shielding purposes. A protective stack of hygienic wipes and moistened towels with an average water thickness of 6.3 g/cm^2^ was evaluated on the ISS and was found to reduce the equivalent dose by 37% [40]. Habitats located on the surface of a planetary body will derive an element of protection even in the absence of an atmosphere or geomagnetosphere. Whereas in free space GCRs are incident from all directions (4π steradians), a planetary body effectively blocks out half of this radiation, with incident radiation only arriving from above (2π steradians or less) [41]. Furthermore, habitats on the surface of the moon or Mars can incorporate local geography (e.g., craters, lava tubes) and materials (e.g., regolith) to provide additional shielding [42,43]. In addition to standard shielding considerations, designs for spacecraft or habitats beyond LEO also envision the creation of a “storm shelter”—a heavily shielded area where crews can take refuge in the event of an SPE. Based on data from previously recorded SPEs, it is estimated that an adequate level of protection can be effectively achieved with an aluminum equivalent of 28 g/cm^2^ [44,45]. While these shelters can mitigate the risks from SPEs, their ability to further augment the risks from GCRs is limited, with even optimal shielding materials estimated to provide no more than a 35% dose reduction [38]. Ongoing developments include research into the use of novel materials such as hydrogenated boron nitride nanotubes, which display promising radiation shielding properties in addition to high strength, thermal resistance, and flexibility [46,47]. Finally, efforts are ongoing to develop an “active shielding” solution—that is, the production of a protective plasma, electrostatic, or magnetic field around the spacecraft that can effectively deflect incoming radiation [48,49]. However, significant technical and engineering challenges remain in this domain, and such approaches are unlikely to be deployed in the near-to-medium term [50].

### 2.2. Transfer Time Reduction

Analysis of the interplanetary and Martian radiation environment by the RAD aboard the Mars Science Laboratory’s Curiosity rover demonstrates that most of the radiation dose was incurred during the transit phase of the mission [26,27]. Follow-up data obtained over the subsequent 2000 sols of the Curiosity rover’s mission on Mars demonstrates a decreased quality factor for GCRs on the planetary surface as compared to free space, with dose equivalent values in the range of 1/3–1/2 of those experienced during transit [51]. Surface habitats can be further augmented with lunar/Martian regolith, whereas options for improving shielding during the cruise phase of the mission are necessarily more limited. Given these challenges, successful efforts to decrease the amount of time spent in interplanetary space are likely to have a significant impact on the total radiation dose incurred during a long-duration mission. Transit time is a less important factor for cis-lunar exploration, where travel time is on the order of 3 d. Based on current chemical propulsion methods, however, any mission architecture to Mars will involve a minimum of 180 d spent in transit during one leg of the journey [28]. Two fundamental design architectures exist for a crewed mission to Mars, based on the trajectory chosen: conjunction-class (long surface stay) and opposition-class (short surface stay) [52]. Conjunction-class missions, which have generally been favored, take advantage of favorable alignment between Earth and Mars during both legs of the journey to minimize propulsion requirements, transit time, and to maximize surface stay (up to 500 d), for a total mission duration of 900–1000 d. By contrast, opposition trajectories limit the overall mission duration to 400–650 d, at the expense of increased transit time, propulsion requirements, and decreased time on the Martian surface (30–90 d). In addition to this, most opposition trajectories involve a Venus fly-by on the return leg of the journey, which brings the perihelion portion of the trajectory within 0.7 AU of the Sun or less, and therefore increases the potential risk to the crew from SPEs [28]. Mission timing with respect to solar cycle will augment the radiation risks during transit, with an increased risk from GCRs and decreased risk from SPEs during solar minimum, and vice versa during solar maximum [30]. Beyond the considerations described above, a meaningful reduction in transit times can only be achieved by advancements in propulsion technology [53]. Nuclear thermal propulsion (NTP), under ongoing development, has the potential to significantly decrease transit times and to make viable new mission architectures [54,55]. While still experimental and not without safety considerations of its own, given the requirement for fissile material on board, NTP nevertheless represents a next-generation propulsion technology that is capable of providing the high thrust and specific impulse requirements necessary for enabling the long-term exploration of Mars and the solar system [56].

### 2.3. Nutritional

Adequate nutrition is critical for human health. Recorded history is replete with stories of traveling parties who succumbed to, or were impacted by, the lack of access to food. Few journeys, however, have proven more physically demanding than space travel. Apart from the provision of required macronutrients, dietary intake during long-duration spaceflight impacts several other areas of astronaut health, including maintenance of the musculoskeletal, visual, cardiovascular, endocrine, and immune systems. As such, the study of food in general, as well as individual dietary agents more specifically, must not be overlooked as a source for radiation countermeasures.

Food is a source of both physical and psychological nourishment for astronauts. Moreover, fruits and vegetables contain phytochemicals, antioxidants, and other compounds known to interfere with oxidative stress pathways, pointing to an important mechanistic basis for countermeasures. The inherent limits of storage space, mass, time, and agricultural resources, restrict crew access to a wide variety of foods. Instead, their options are limited to a set menu of single-serving, prepacked, shelf-stable meals, with an even more limited ability to bring select personal favorites that need to be consumed soon after launch. Moreover, while resupply vehicles currently deliver fresh stores of food and other comforts of home to LEO several times per year, this will no longer be feasible as we venture farther from Earth. Much of the literature related to nutrition in space has focused on the *macro*-nutrient value of food needed to support caloric expenditure as a means of sustenance, as well as its preservation. This section will instead focus on the *micro*-nutrient value of foods and dietary agents, and their ability to act as radiation countermeasures.

Radiation-induced oxidative damage has been shown to play a role at all levels of the human body [57]. Oxidative stress induces molecular alterations that can lead to mutation, neurocognitive (NC) decline, increased risk of epithelial cancers and cardiovascular disease, decreased resilience to infections, and numerous additional health effects often referred to as ‘premature aging.’ Reactive oxygen species- (ROS-) derived damage is well-documented during space flight, with radiation exposure thought to be the primary culprit [58,59,60,61,62,63,64,65,66,67].

**Figure 1:**
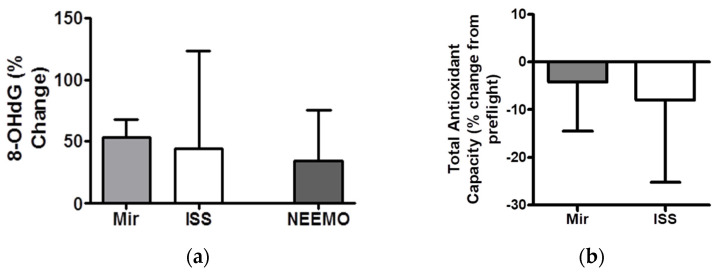
(**a**) The percent change of 8-hydroxy 2′deoxyguanosine (8-OHdG) from pre-flight values for Mir (*n* = 2), International Space Station (ISS) (*n* = 11), and the ground based analog NEEMO (*n* = 6) (Smith et al. 2004). (**b**) Total antioxidant capacity after space flight for Mir (*n* = 2) and ISS (*n* = 11). [59,64].

Plasma malondialdehyde (MDA), 8-iso-prostaglandin F2α (PGF2), and urinary 8-hydroxy-2′deoxyguanosine (8OHdG) have been measured during and after the flight as indicators of lipid peroxidation (MDA and PGF2) and DNA damage (8OHdG) [58,59]. Several investigations, including data from Mir, Skylab, and the Longitudinal Study of Astronaut Health, show a significant elevation of urine 8OHdG after long-duration missions (Figure 1a) and/or missions beyond LEO. Likewise, these missions have also directly measured Total Antioxidant Capacity (TAC) (Figure 1b). Perhaps unsurprisingly, these same results were not found after short-duration missions [58,59,60,61,62,63]. Interestingly, however, these results are supported by data from the NASA Extreme Environment Mission Operations (NEEMO), where crewmembers underwent a 14-d saturation dive with increased partial pressure of oxygen [64].

Another means to assess oxidative stress is to look at the quantity of constituent oxidative stress protection molecules pre- and post- flight. Examples include superoxide dismutase, glutathione reductase, selenoprotein family (P, W, V, S,) glutathione peroxidase, and thioredoxin reductase (Table 1); the latter three of which require nominal selenium levels for complete enzymatic function [68].

**Figure 2:**
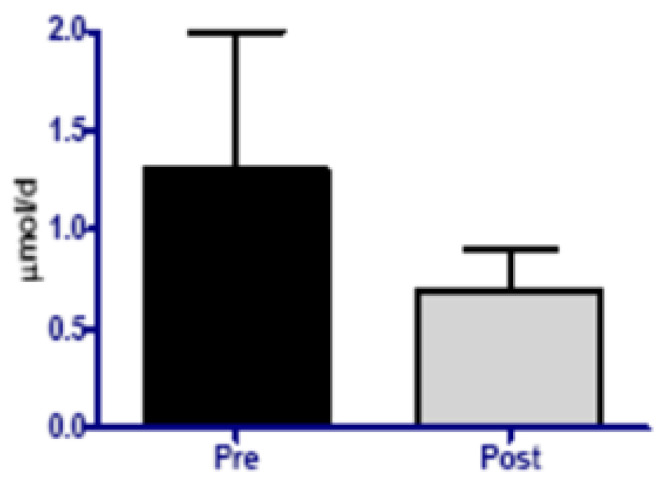
Urine Se values pre- and post-flight in ISS crew members [73].

Selenium seems to be one of several micronutrients that may be depleted during long-duration spaceflight and could affect oxidative damage defense (Figure 2). Selenium deficiency has also been associated with impaired function of the immune system [68]. Moreover, supplementation in individuals who are not selenium-deficient appears to stimulate the immune response. In two small studies, healthy [69,70] and immunosuppressed individuals [71] supplemented with 200 mcg/day of selenium for eight weeks showed an enhanced immune response to foreign antigens compared with those taking a placebo. A considerable amount of basic research also indicates that selenium plays a role in regulating the expression of cell-signaling molecules called cytokines, which orchestrate the immune response [72].

The presence of radioprotective dietary compounds at the time of exposure may lessen the initial ionizing events from radiation, such as cell death and induction of mutations. Radioprotective agents have been studied in the context of military (nuclear warfare) and medical (radiation oncology) applications. In terms of effectiveness, the ideal radioprotective agent must be present at the time of exposure, close to the site of damage (remaining present until the damaging stimuli have ceased), and able to protect against injury or damage arising from multiple pathways [74]. In practical terms, the ideal agent would be deliverable orally; it would be effective for days to weeks (or could be given repeatedly); it would have minimal effects on performance; it would have no cumulative toxicity and minimal side effects; it would be compatible with other drugs that may need to be administered concomitantly, and; it must have a long shelf life. Although hundreds of potential radioprotectants have been studied to date, only food or food-derived compounds seem likely to be able to meet all of the above criteria.

Many chemoprotective compounds are present in natural sources (Table 2). Indeed, it is preferable to derive these agents from natural sources, both for palatability and for the quality of their biological activity. However, as stated earlier, it may not always be possible to provide fresh foods and vegetables for an entire mission (especially long-duration missions), and other means of accessing these nutrients, particularly with regard to their chemoprotective qualities, must therefore be developed. Dietary supplements in compressed tablet form or other alternative delivery mechanisms with high shelf life, stability, and storage efficiencies may prove to be worthy solutions to this dilemma.

It has been reported that such agents found in the diet have the potential to decrease the types of oxidative damage observed among individuals exposed to ionizing radiation [76,77,78,79,80,81,82,83,84,85,86,87,88,89,90,91]. Unfortunately, most known exogenous radioprotective or so-called antioxidant compounds are usually needed in very large doses in order to demonstrate clinical efficacy as single agents. These dosages often exceed tolerability and/or safety levels. Moreover, oxidative damage is the result of multiple complicated biochemical pathways that can seldom be corrected by one single intervention or one single compound. The use of single dietary antioxidant agents may alter or down-regulate of other natural antioxidant defense mechanisms [77]. Thus, it is postulated that a countermeasure formula mixing safe intake levels of each of the most effective dietary chemopreventive molecules may allow delivery to humans without the toxicities associated with high-dose, single agents.

As part of a broader effort to pursue exploratory missions in 2005, NASA entered into a Space Act Agreement with industry partners to research dietary supplement agents, and formulate combinations as a means to promote astronaut health, increase physical and mental resiliency, and act as potential countermeasures against space-induced oxidative stress. A number of experiments and publications have since taken place as a result of these joint efforts [92,93,94,95,96]. The radioprotectant protocol developed as a result is comprised of a daily multi-vitamin and multi-mineral formulation intended to ensure and standardize the intake of all micronutrients identified as essential, plus a combination of food-based polyphenols, carotenoids, flavonoids, amino-acid derivatives, and other compounds shown to have antioxidant activity, or provide some measure of chemoprotection against ROS (Table 3).

Independent research to assess the viability of dietary agents, supplements, and combinations, in promoting human adaptation and reducing the damaging effects of ionizing radiation has also been undertaken [92,93,94]. Investigators at the Northern Ontario School of Medicine (NOSM) and associated institutions led by Dr. Boreham et al. [40,41,42,43,44,45,46] have demonstrated DNA-protectant effects, amelioration of both age-related and radiation-induced cognitive decline, longevity extension following exposure to radiation, and positive modulation of the endogenous nitrative and oxidative stress response, following ingestion of a dietary radioprotectant formula. Others [95,96] have similarly tested and successfully used dietary radioprotectants in cell and animal models, demonstrating a degree of universality in this approach to effectively address a range of ROS-mediated issues. Select examples are listed below:

#### 2.3.1. Neuro-Cognitive Decline

**Figure 3:**
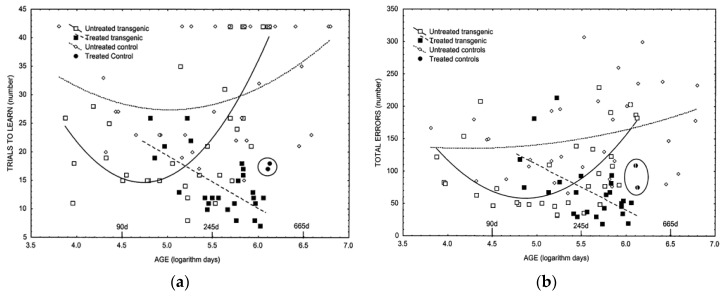
(**a**) Number of trials required for transgenic mice (TGM) and age-matched normal controls to learn an eight-choice maze across lifespan. Regression lines had the following equations: untreated TGM (y = 13.37(x^2^) − 126.54(x) + 313.96); untreated normal mice (y = 3.72(x^2^) − 36.92 (x) + 118.76) and treated (antiaging supplement) TGM (y = −9.19(x) + 65.49). Age in days is indicated above the *X*-axis. (**b**) Total errors committed during learning an eight-choice maze by TGM and normal controls across lifespan. Regression lines had the following equations: untreated TGM (y = 77.19 (x^2^) − 755.66(x) + 1888.15); untreated normal mice (y = 6.7(x^2^) − 47.15(x) + 206.74) and treated (antiaging supplement) TGM (y = −70.01x + 459.76). Age in days is indicated above the X axis. Data from Lemon et al. [97].

Research carried out by Dr. Lemon et al. [97] on rodents demonstrated a complete abolishment of any signs of cognitive decline following the administration of a dietary radioprotectant formula on transgenic mice (TGM) known to have elevated and progressively increasing free radical processes in brain tissues, which culminates in rapid age-related loss of cognitive ability (Figure 3a,b).

The dietary agents effectively promoted membrane and mitochondrial integrity, increased insulin sensitivity, reduced reactive oxygen and nitrogen species, and ameliorated inflammation. Further research [98] on transgenic mice known to experience a greater than 50% loss of cells in key regions of the brain, severe reductions in brain metabolism, and impaired blood flow resulting in marked cognitive impairment, demonstrated that the dietary radioprotectant formula abrogated the severe cell loss. It also protected brain metabolic rate and improved blood perfusion, presumably increasing brain oxygenation. This highlights the potential for prevention or amelioration of human neuro-pathologies that are similarly associated with oxidative stress.

**Figure 4:**
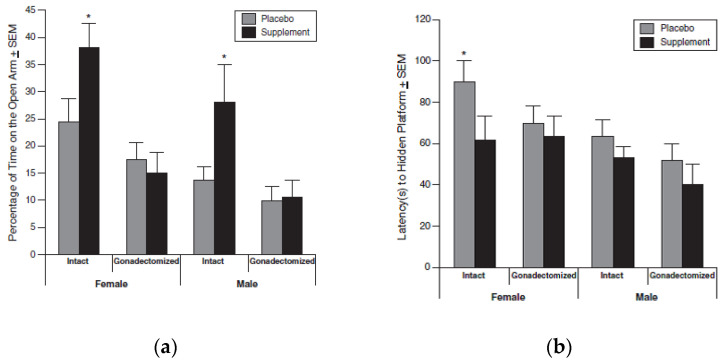
(**a**) The percentage (mean ± SEM) of time (during a 300 s task) spent on the open arms of the elevated plus-maze for intact and gonadectomized, female and male rats administered placebo (grey) or supplement (black). A * indicates a significant interaction of gonadal status and supplement condition to be greater for marked groups. (**b**) The mean (± SEM) latency (s) to the hidden platform in the water-maze for intact and gonadectomized, female and male rats administered placebo (grey) or supplement (black). A * indicates a significant difference for the marked group to be greater compared to all other groups. (Reprinted from [90] with permission from Elsevier).

Friedman and Frye [90] separately conducted a series of experiments on Long–Evans rats, in order to assess the anti-anxiety, cognitive, and steroid biosynthetic effects of a similar multiple dietary agent protocol. Intact rats supplemented with the countermeasure exhibited greater anti-anxiety behavior in the elevated plus maze. The countermeasure also enhanced visual-spatial performance of all rats in the Morris water maze and increased levels of 5α-androstane,17ß-diol-3α-diol (3α-diol) in the hippocampus (but not other brain regions) of females. Thus, the diet altered anxiety, cognitive behavior, and brain production of steroids. Both the anti-anxiety and improved cognitive performance effects were more pronounced in rats with an intact reproductive axis (Figure 4a,b). There were neither effects on sexual behavior, nor evidence for trophic effects on any of the observed tissues of the supplemented group. These results coincide with and validate the cognitive impact observed by Lemon et al. [98].

#### 2.3.2. Physical Endurance

Experiments to assess spaceflight-related muscular fatigue reduction following the ingestion of oral dietary antioxidants—primarily consisting of N-acetylcysteine, but also combined with other micronutrients, were conducted on crewmembers training in the Neutral Buoyancy Laboratory (NBL) during 6-8 h hyperoxic environmental exposures [99]. Muscle fatigue is a known sequela of oxidative stress, and it is believed to be aggravated by the pro-oxidative environment present in space. The ability to perform tasks observed to induce forearm fatigue during extravehicular activities (EVA) was evaluated as follows. (A) The magnitude of oxidative stress in EVA crewmembers, as measured by markers of lipid peroxidation, DNA damage, and total oxidant capacity, along with the efficacy of dietary agents in reducing simulated EVA-induced total oxidative stress; and (B) The efficacy of an antioxidant countermeasure to reduce muscular fatigue seen during EVA-type activities. The molecular biomarkers and forearm endurance measures were both markedly improved in the nutritional countermeasure group when compared to placebo [99].

#### 2.3.3. Longevity and Reduction in Carcinogenesis

A complex radioprotectant dietary supplement was shown to be effective at reducing tumorgenesis (30%), carcinomas (67%), metastasis (100%), and the occurrence of multiple primary tumors (74%) in cancer-prone heterozygous Trp53+/-mice [100]. Reduction of pulmonary adenocarcinoma (62%) was of particular note given that lung cancer is the second leading cause of death in humans. Tumors showed pronounced age-related expression in untreated animals older than 600 d. Benefits of treatment only emerged in these later ages, suggesting that the formula acted on mechanisms common to aging and cancer. Although longevity was not statistically different between treatments in this particular study, longevity was strongly related to the compliance of mice in eating the supplement. Linear regression revealed a strong positive relationship between the proportion of supplements eaten and the longevity of mice within the treatment group. In addition, other studies have reported that the longevity of both TGM and normal mice is extended by this dietary supplement approach [75]. Treated TGM showed a 28% increase in mean longevity. An 11% increase in mean longevity was also significant for treated normal mice, compared to untreated normal mice [75].

**Figure 5:**
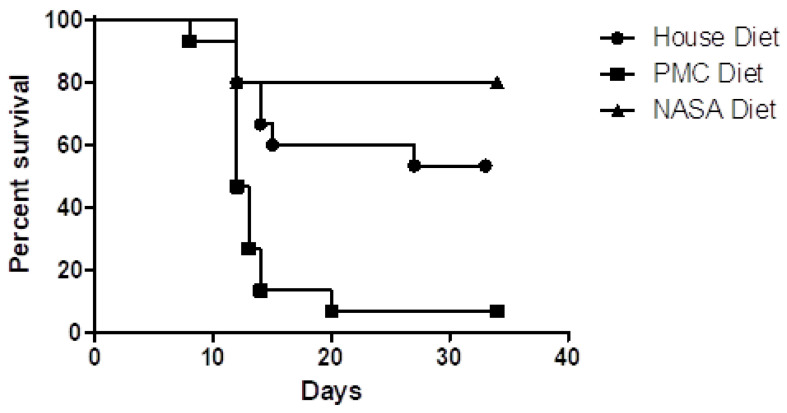
Rodent survival after 9.5 Gy gamma-ray exposure, with 80% of rodents surviving 20 days that received the “NASA diet” (e.g., chow with proposed low-dose multi-agent nutritional supplement), vs. <60 for standard “House Diet” (e.g., chow alone without any supplement), and <20% survival for “PMC Diet” (e.g., chow with high-dose antioxidant supplements) [101].

A preliminary study that included the radioprotectant formula was conducted at the University of Pittsburgh and published by Dr. Epperly et al., as part of a broader evaluation of different countermeasures in the protection of rodents exposed to LD 100/15 total body γ-radiation (9.5 Gy) in terms of acute survival. The animals were fed the group-specific agents for 2 weeks prior to irradiation (Figure 5) and followed until expiration, at which time they were autopsied. Body weight and health parameters for supplemented groups compared to un-supplemented/placebo (e.g., house diet.) were assessed. There were no adverse health or weight effects observed in the supplemented diet animal group as compared to placebo. Furthermore, groups supplemented with the radioprotectant “NASA” diet exhibited the greatest degree of acute survival as compared to other dietary agents or placebo.

**Figure 6:**
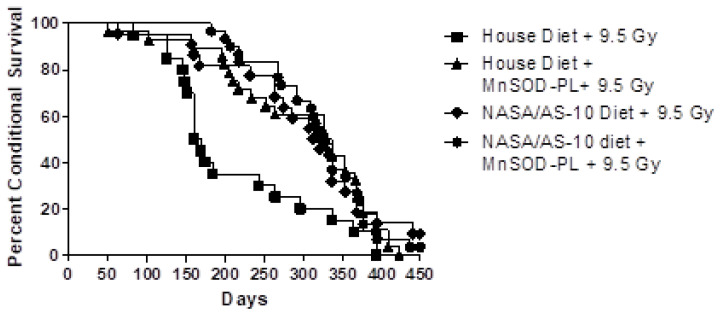
Survival curves for each of four test groups receiving 9.5 Gy total body irradiation. Conditional survival of mice on the antioxidant diet was significantly improved over the 450 days of observation compared to that of those on the house diet. Data from [101].

A follow-up investigation of the countermeasures’ late-effects and long-term survival potential was carried out by the same team [101]. Mice were once again segregated into groups; exposed to 9.5 Gy of total body irradiation and followed through to expiration. Epperly et al. observed that conditional survival of mice on the radioprotectant formula was significantly improved over the 450 days of observation compared to those on the placebo “House Diet”. Furthermore, rodents on the radioprotectant formula and a pharmaceutical radioprotectant, alone or in combination, showed an overall improvement in conditional survival compared to those on the placebo diet (*p* = 0.010) (Figure 6).

These results establish that antioxidant diet supplements ameliorate radiation-induced life-shortening and provide support for the concept of continuing oxidative stress in the post-irradiation cellular microenvironment of tissues, organs, and organ systems. Moreover, they provide a window into the potential for synergistic activity of dietary and pharmaceutical countermeasures, when taken concomitantly.

Animal models on the dietary radioprotectant formula regimen had significant increases both in survival as well as performance, compared to animals on a standard diet [101]. Evidence suggests that a multi-faceted approach that takes into account a variety of lower dose oxidative stress modulators across several biochemical pathways, including dietary antioxidants, taken in combination, may provide more effective protection while ameliorating many of the safety and toxicity concerns associated with large intakes of these same compounds. A dietary formula approach may be enhanced by targeted pharmaceuticals for acute high-dose exposures.

Additional research is currently being carried out on novel dietary ingredients that may play a role in longevity, and could potentially enhance the effects of these dietary radioprotectants. Alpha-Ketoglutarate (AKG), for example, is a carbon and nitrogen metabolism regulator that has been found to extend healthy lifespan, and reduce morbidities in animal and human models [102,103,104,105]. NAD+ precursors such as Nicotinamide Mononucleotide (NMN) and Nicotinamide Riboside (NR) have been shown to be effective at replenishing NAD+ levels and cellular function [106]. They are also being investigated for their ability to activate sirtuins, which play a role in cellular longevity. Other supporting nutrients currently under consideration include the aliphatic polyamine Spermidine, and the vitamin-like methylation cofactor Trimethylglycine (TMG). Spermidine is known to play a role in ribosomal function, RNA transcription, and inhibition of NOS. Spermidine may enhance longevity through autophagy, inflammation reduction, lipid metabolism, and regulation of cell death [107,108,109,110]. These novel dietary compounds show promise as coadjuvants to any radioprotection formula, by acting as longevity modulators and cellular metabolic regulators.

It should further be noted that although some dietary agents may generally be considered radioprotectants, their role is often mired with complexity. For example, turmeric-derived curcuminoids and grape-derived resveratrol are often thought of as antioxidants. Yet recent research has shown their dual role as both protect healthy cells and also sensitizing neoplastic cells so as to make them vulnerable to radiation-induced death [111,112]. Such a dual and seemingly conflicting role of micronutrients should be further evaluated during the development of a space use countermeasure.

#### 2.3.4. Epigenetics, Ophthalmic Change, and Impact on Visual Health

Several physiological changes are known to take place in the eye during spaceflight. Their severity and impact are dictated by a number of factors, including mission duration and exposure to radiation. Analysis of NASA’s Longitudinal Study of Astronaut Health (LSAH) has demonstrated an increased incidence and early appearance of cataracts among the astronaut population [113]. Cucinotta et al. postulate that this is a result of chronic exposure to the space radiation environment.

Spaceflight-associated neuro-ocular syndrome (SANS) describes a series of morphologic and functional ocular changes in astronauts first reported by Mader and colleagues in 2011. SANS is clinically defined by the development of optic disc edema during prolonged spaceflight. However, as our understanding of the ocular changes evolves, the definition of SANS is expected to evolve in turn. Other ocular SANS signs that arise during and after ISS missions include hyperopic shifts, globe flattening, choroidal/retinal folds, and cotton wool spots. Over the last 10 years, approximately 1 in 3 astronauts flying long-duration ISS missions have presented with SANS [114]. The predominant theory used to explain these phenomena centers on microgravity-induced fluid shifts within the human body, leading to increased intracranial pressure, which ultimately impinges on the optic nerve or the eye itself. While SANS itself is not believed to be the result of radiation exposure, it predisposes an already vulnerable organ to further oxidative stress, which can thus be aggravated by the space radiation environment.

According to Smith et al. [115], biochemical evidence documented changes in circulating metabolites of the one-carbon metabolism pathway (including homocysteine and methylmalonic acid to assess vitamin B12 status) in astronauts with vision issues. These biochemical differences existed before flight [116]. Furthermore, preflight circulating concentrations of serum folate, vitamin B6, vitamin B12, and some of the one-carbon intermediates were related to the change in refraction observed after long-duration space flight [116]. Follow-up testing to evaluate the incidence of polymorphisms in the one-carbon metabolism pathway in astronauts, and to relate these to vision changes and related physiology, has recently yielded advancements in our understanding of the role that these nutrients play on enzyme polymorphisms and epigenetic changes.

Both B-vitamins and genetics were significant predictors of many of the ophthalmic outcomes observed in crew members. Specifically, every astronaut with the minor form of the MTRR single-nucleotide polymorphism (SNP) developed some ocular issue; but not every astronaut with some ocular issue had this form of the SNP. B-vitamin nutritional status indicators often significantly improved model fit. Whether vitamin supplementation can override genetics, alter biochemistry, and counteract the response to the causes of SANS in at-risk individuals needs to be further evaluated. However, the evidence thus far suggests that genetics may predispose some individuals to develop ocular pathologies, but B-vitamin status may impact their epigenetic expression, and impart some level of protection to the eye.

#### 2.3.5. Safety and Tolerability

Following the above animal results, Montesinos et al. [117] conducted a clinical trial to evaluate the safety and toxicity of the radioprotectant formula in healthy human volunteers. This multi-center, randomized, double-blinded, placebo-controlled trial of approximately 187 subjects assessed the acute and chronic safety, as well as potential clinical outcomes following ingestion over a 3-month period, with a subgroup followed for a total of 6 months to assess adverse events. Preliminary results of this trial showed that adverse events were few (<7%) and mild. During the initial 3-month group, only 3 subjects (1.7%) experienced events that were severe enough to discontinue intake, and only one was potentially attributable to the agent. A secondary cohort of volunteers was followed up for an additional 3 months, for a total of 6 months of intervention. The follow-up group did not report any instance of adverse events during the second 3-month period (0%). The nutritional protocol was well-tolerated overall.

### 2.4. Pharmacologic

#### 2.4.1. Pharmaceutical Radioprotectors

When entering an environment with a known risk of exposure to ionizing irradiation, radiation protectors may be given. These are pharmacologic agents and include small molecules, cytokines, and novel radiation exposure modulators that can be administered prior to known radiation exposure [118]. In contrast, radiation mitigators are those agents given after radiation exposure, but prior to the detection of symptoms or signs of radiation disease. The window for administration of radiation mitigators depends upon the quality of the radiation beam, dose, and percent of the body exposed [118].

The first studies of radiation protectors included those focused on delivering agents, that would modulate a known ionizing irradiation event in cells, tissues, organs, and organisms. The production of ROS was known to lead to nuclear DNA double-strand breaks, which were directly associated with the degree of radiation damage [6,57]. Also important for assessing irradiation damage was the role of volume of tissue or organ exposed, the dose rate of the irradiation beam, and quality of the radiation beam whether restricted to gamma rays (x-rays), beta irradiation (electrons), alpha particle irradiation, or high linear energy transfer (LET) ionized particles [6]. This latter category includes doses associated with galactic cosmic radiation (GCR) [45,118]. Radiation protectors utilized in clinical radiotherapy, as in the treatment of cancer patients, rely upon data from animal model systems in which all the parameters of irradiation exposure can be precisely quantitated and administration can be controlled [45]. In contrast, space irradiation can be sporadic by nature, particularly when involving SPEs. Therefore, a major challenge for the development of radioprotectors for use in the space program is the need for agents that have applicability across multiple exposure scenarios which are safe for multiple or even continuous administration. It should be noted that several dietary agents may offer some radioprotective action, creating an inevitable overlapping between pharmacologic and nutritional countermeasures sections. Nevertheless, given the nature of nutritional countermeasures, and the fact that they can be either incorporated with or sourced from foodstuffs, this section focuses solely on pharmacological radioprotectors.

##### Strategy for Use of Radiation Protector Pharmacologic Agents

The mechanism of action of a radioprotector must be clearly defined. A major development in defining the mechanism of action came with the discovery of the importance of mitochondria in ionizing radiation damage.

In the 1970s, assays for detection of DNA double-strand breaks (sucrose gradient centrifugation techniques to size DNA fragments), led to the conclusion that ionizing irradiation damaged cells in a direct dose-response relationship based on the number and density of DNA double-strand breaks [119,120,121]. Accordingly, radioprotector drugs were fashioned to prevent DNA double-strand breaks. Radical oxygen species (ROS) produced by irradiation were known to cause DNA double-strand breaks, thus, drugs designed to neutralize ROS became the focus of research. ROS scavenger drugs included sulfhydryl compounds and antioxidants. Administration of these agents by intravenous or topical 4-hydroxy-2,2,6,6-tetramethylpiperidinyloxy (TEMPO) to ameliorate radiation skin burns was a target for clinical development. A major breakthrough was the discovery of the role of mitochondria in radiation damage. Nuclear DNA double-strand breaks were known to be repaired within minutes after irradiation exposure, and subsequent events leading to lethality were shown to have cell transport from the nucleus to mitochondria of pro-apoptotic molecules including BCL-XL, p21, p53, and others [119,120]. Nuclear to mitochondria transport was shown to lead to consumption of mitochondrial antioxidant stores, leakage of cytochrome C from its natural role in electron transport oxidative metabolism, and the transformed role of cytokine C into a peroxidase, which leads to the production of more oxidative stress and greater cell damage [121]. Understanding the role that mitochondria played in irradiation-induced cell death, focused drug development to agents that targeted the mitochondrial membrane to stabilize the changes leading to cytochrome C separation from its “protective” lipid cardiolipin. Potent radiation protectors were designed based on the principle of mitochondrial targeting of antioxidants [122,123]. These included delivery of drugs by mitochondrial targeting signals including documents of the antibiotic hemi-gramicidin, or triphenyl-phosphonium, both of which led to an increase in the concentration of an antioxidant small molecule in the mitochondria, and proportional radiation protection.

##### Multiple Pathways of Cell Death

Apoptosis was the first cell death pathway associated with ionizing irradiation. Several other cell death pathways have been reported to be involved in ionizing irradiation cell killing: These include necroptosis, ferroptosis, parthanatos, pyroptosis, and multiple other “secondary cell death” inducing pathways, such as the increased concentration of inflammatory cytokines and inflammatory cells causing vascular destruction and tissue swelling. Secondary cell death mechanisms can result in the activation of one or more of the biochemical pathways associated with cell death [119,120]. The discovery of multiple cell death pathways in irradiation damage led to the discovery of drugs targeted to each of these pathways, some of which could block the signaling in more than one pathway. A sequence of activation of cell death pathways was not uniform and some were activated later, such as the delayed reaction of necroptosis by TNF-α, which required 24–48 h after radiation exposure to reach levels associated with the initiation of this death pathway [120].

Important for space irradiation is the requirement to determine whether small molecule inhibitors of gamma irradiation-induced cell death by each of these pathways also apply to the inhibition of proton or GCR-induced cell death. It is also important to determine the effectiveness of a radioprotector against neutron irradiation, which would arise from GCR interaction with the shielding components of the spacecraft. Therefore, knowledge of the cell death pathways and the discovery of drugs to target each pathway is only the first step. In an ideal case, a drug should be effective against all forms of irradiation (beam quality, dose rate, and sporadic nature of exposure) [6,119].

##### Polypharmacy and Radiation Protection

To be effective radioprotector drugs, given all the above considerations, the role of polypharmacy must be considered. Astronauts may be required to take medication for other non-radiation-related indications such as infection or physical injury [6,119]. The safety of each radioprotector drug should be documented in each of these settings, as well as in the setting of combined injury. Radioprotector drugs must be safe in the setting of other challenges to human physiology including infection, trauma, fracture, and thermal burn. Higher doses of radioprotector drug may be necessary for a setting of combined injury [6,123]. Some forms of combined injury may elevate or exacerbate the same cellular death pathways involved in irradiation cell killing and necessitate modifying the dose of a radioprotector to be administered. Space radiation protectors and mitigators will likely also include the use of novel orally administered second-generation probiotics and other nutraceuticals [124].

#### 2.4.2. Managing Higher Radiation Exposures

Measures to manage Acute Radiation Syndrome (ARS) may already be in the exploration medical kit to include anti-emetics and IV or IO hydration for nausea, vomiting, and anorexia; antibiotics in the case of granulocytopenia-induced endogenous bacterial infections [125].

The primary risk from space radiation exposure will be long-term excess cancer, cardiovascular and neurodegenerative morbidity, and mortality [125]. However, several design reference missions, especially for the long stay on the Martian surface, involve the utilization of mid-size radioisotope thermoelectric generators (RTG), containing e.g., Plutonium-238 (primarily alpha emitter) or Americium-241(primarily gamma-emitter) for surface power of the habitat. Some transit vehicle designs employ nuclear thermal or nuclear electric propulsion systems, possibly with electric ion or plasma drives. Accidental exposures to the crew from these nuclear devices could reach levels that may produce ARS, so developing a risk mitigation plan for the level of exposure that could produce APS or ARS is reasonable [125].

Acute radiation disease (ARD) or acute radiation syndrome (ARS) are defined as the collective toxic clinical states observed from the acute immunological and pathological processes in various doses of irradiated mammals; to include: radiation systemic inflammatory response syndrome (RSIRS), radiation toxic multiple organ injury (RTMOI), radiation toxic multiple organ dysfunction syndromes (RTMODS), and finally, radiation toxic multiple organ failure (RTMOF). This typically may occur with acute exposures in excess of 4 Sv in 24–48 h. This type of exposure for all the crew may be beyond the capacity of the exploration medical kit and the crew medical officer to alleviate. If it were to occur in a single crew member, in addition to the above medical measures for APS; the crew member could be rescued by autologous bone marrow or hematogenous stem cells, stored frozen in a radiation-protected location, and donated by each crewmember prior to flight [125].

Both high dose and high dose rate radiation induce apoptotic vs. necrotic cell death in radiosensitive cells, with the formation of radiation-induced toxins—proteins, enzymes, peptides, and their subsequent induced acute inflammatory processes. Radiation necrosis is the most severe form of radiation-induced injury, and when widespread, has challenging therapeutic implications.

### 2.5. Immunomodulation

The development of radiation protection agents based on immunomodulation* is one of the more promising fields. These include prospective DNA and RNA-based antiradiation vaccines; as well as antiradiation antidotes—a group of neutralizing, therapeutic antibodies and natural inhibitory proteins which suppress inflammation, pathological apoptosis, and necrosis of irradiated tissues [126,127,128,129].

The role and efficacy of antiradiation antidotes are based on fast and short-acting suppression of pathological immune reactions induced by high, medium, or small doses of radiation [130]. Conversely, the role and efficacy of prospective antiradiation vaccines (DNA antiradiation vaccine or RNA antiradiation vaccine) are based on immune-prophylaxis and prevention of acute pathologic autoimmune inflammatory reactions.

* Refer to Appendix A for a comprehensive discussion on the impact that space radiation exposure has on the immune system.

#### 2.5.1. Antiradiation Antidote with Suppression Activity of Membrane Attack Complex (MAC)

Activation of complement systems and proteolytic cascades are a natural result of radiation exposure, in which serine proteases are activated from each other by proteolysis. Depending on the ionizing radiation (IR) dose, uncontrolled complement activation contributes to the development of ARS. Immunomodulation with inhibition of the complement serine proteases shows promise as an attractive therapeutic strategy [131].

Antiradiation serine inhibitors, such as serpins and macro-globulins, undergo significant conformational change during the inhibitory process of radiation-activated MAC Cascade. Serpins and macro-globulins form irreversible complexes with the target proteases. Other groups of small protein inhibitors of serine proteases can form reversible complexes with the target protease [131]. Serpins are known for their unusual mechanism of action. Unlike the common competitive mechanism of protease inhibitors that bind to protease active sites and block their entry, serpins irreversibly inhibit the function of their protease by undergoing large conformational changes to disrupt the active site of it [131].

Members of the α2M group have been identified in a wide range of vertebrate and invertebrate species and comprise the C3, C4, and C5 components of the vertebrate complement system. They are the largest major non-immunoglobulin protein in human plasma. α2Ms are thought to be early innate immune components, similar to opsonin, but their role in the proteolytic attack of invading pathogens remains hypothetical. alpha-2-Macroglobulins (α2-Ms) act as an anti-protease, inhibit fibrinolysis by inhibiting plasmin and kallikrein, and, therefore, inactivate an enormous variety of proteinases [131].

Antiradiation action of small protein inhibitors of serine proteases is based on their ability to form reversible complexes with the target protease by thermodynamic terms [131]. Immuno-modulation of acute immune reaction developed in an irradiated mammal includes inhibition of MAC synthesis. MAC synthesis is inhibited by MAC-inhibitory protein or “protectin”, or in some cases could be named as “antiradiation protectin”. CD59 is found on the surface of normal human cells. CD59 is an example of a glycosyl phosphatidyl-inositol (GPI)–linked protein [131]. Its function is to protect normal human cells from being accidentally destroyed by their own autoimmune MAC. CD59 prevents polymerization of C9 by the complex C5b–C6–C7–C8, thus preventing synthesis of MAC on normal cells. In a rare human condition known as paroxysmal nocturnal hemoglobinuria, erythrocytes lack CD59 and so can be lysed by MAC [132].

#### 2.5.2. Antiradiation DNA and RNA Vaccines

A great deal of effort has been devoted to the development of Antiradiation DNA vaccine-based immunotherapies for the treatment of ARS, as well as the autoimmune and inflammation components of ARS. Prevention or inhibition of massive death of cells via apoptosis/necrosis has been achieved, as well as radiation-induced DNA damage such as double-stranded DNA breaks (DSBs) and single-stranded DNA breaks (SSDs) [128,133,134,135,136,137,138,139].

The DNA damage response (DDR) rapidly recognizes DNA lesions and initiates the appropriate cellular programs to maintain genome integrity. This includes the coordination of cell cycle checkpoints, transcription, translation, DNA repair, metabolism, and cell fate decisions, such as apoptosis or senescence [128,133,134,135,136,137,138,139]. DSBs are induced by exogenous DNA breaking agents or endogenous reactive oxygen species, and the rearrangement of antigen receptor genes in the adaptive immune system [128,133,134,135,136,137,138,139].

ATM serine/threonine kinase, symbol ATM, is a serine/threonine-protein kinase that is recruited and activated by DNA double-strand breaks. It phosphorylates several key proteins that initiate activation of the DNA damage checkpoint, leading to cell cycle arrest, DNA repair, or apoptosis. Several of these targets, including p53, CHK2, BRCA1, NBS1, and H2AX are tumor suppressors [128,133,134,135,136,137,138,139].

Cells respond to DNA damage by activating checkpoint pathways that delay progression through the cell cycle. This cell cycle delay provides the necessary time for the cell to assess and repair the damage before reentering the cell cycle. If the damage is determined to be beyond repair, the cell may undergo apoptosis to prevent mutations from being propagated [128,133,134,135,136,137,138,139].

When mammalian cells are exposed to IR or radiomimetic drugs, a signal transduction pathway is activated that arrests cells in the G1, S, and/or G2 phases of the cell cycle. The G1 arrest is the best characterized and is dependent on a functional p53 response that leads to transcriptional activation of the G1-specific cyclin-dependent kinase inhibitor p21/WAF1/CIP [128,131,133,134,135,136,137,138,139,140].

The ATM-associated DNA-dependent protein kinase activity suggests a mechanism by which ATM or an associated subunit can detect DNA damage and activate an associated protein kinase that phosphorylates substrates that lead to cell cycle arrest [139]. ATM protein levels were found to vary considerably among different cell lines, ranging from undetectable in many AT cell lines and HL-60 cells to high levels in HeLa, U2OS, and normal human fibroblasts.

Although cells with no detectable ATM (AT cell lines and HL-60) are extremely sensitive to IR, the steady-state protein levels of ATM (measured per cell or per milligram of total protein) did not correlate with radio-resistance as measured by the surviving fraction at 2 Gy [139,141,142,143,144]. Overexpression of the ATR kinase domain can also correct radiosensitivity in AT cells [139] Amongst lesions induced by ionizing radiation, double-strand breaks (DSBs) are the most lethal type, which could lead to cell apoptosis or necrosis [132].

To repair DSBs, two main DNA damage repair (DDR) pathways, homologous re-combination (HR) and non-homologous end-joining (NHEJ), are activated. These two main DSBs repair pathways play an indispensable role in initiating and processing cancer. Patients harboring deficiencies affecting HR or NHEJ often present increased susceptibility towards cancer [127,145,146,147].

Previous results demonstrated the efficacy of the anti-radiation DNA vaccine for immune-prophylaxis and immune-therapy of acute radiation syndromes with optimization of clinical futures and critically improving lethality rate [127]. Authors propose that DNA isolated from antigen-presenting cells, separated from blood and lymph of irradiated mammals, plays an important role in the activation of the adaptive immune system, expression, and up-regulation of ATM serine/threonine-protein kinase, and development of significant radio-resistance to external radiation.

#### 2.5.3. Immunomodulation of Activity of B-cells after Irradiation

B cells are key players in the adaptive immune response. They are potent antigen-presenting cells that can produce both pro- and anti-inflammatory cytokines and have the capacity to generate terminally differentiated antibody-secreting plasma cells [141,148,149,150,151,152,153,154].

Thus, B cells represent important targets for the treatment of multiple autoimmune disorders, for the induction of graft survival, or for the treatment of skin and lung fibrosis, and can act as powerful modulators of tissue regeneration [141,148,149,150,151,152,153,154]. Mesenchymal stromal cells (MSC) from the amniotic membrane of the human term placenta (h AMSC), and the conditioned medium generated from their culture (CM-h AMSC) offer significant tools for their use in regenerative medicine mainly due to their immune-modulatory properties [154].

MSC h AMSC and their CM have been successfully exploited in preclinical disease models of inflammatory and autoimmune diseases where depletion or modulation of B cells have been indicated as an effective treatment, such as inflammatory bowel disease, lung fibrosis, would healing, collagen-induced arthritis, and multiple sclerosis [154].

Potentially MSC from h AMSC can be an effective therapeutic agent for the treatment of acute radiation diseases and chronic radiation disease.

#### 2.5.4. Immunomodulation of Cytotoxic Activity of T-cells after Irradiation

Effector CD8+ T cells, also commonly known as cytotoxic T lymphocytes (CTLs), play a key role in cellular toxicity and, when properly activated, are able to effectively destroy irradiated cells.

The aims of this study were to obtain CD8+ CTLs specific for the epitopes, which are characteristic to assess the cytotoxic activity after irradiation, and elaborate methods of inhibition of CD8 T cells cytotoxicity.

For radiation-inducing cytotoxic activity of CD8+ T cells through the release cytotoxic proteins, the principal mechanism through which cytotoxic T cells act is by the calcium-dependent release of specialized lytic granules upon recognition of antigen on the surface of a target cell. These granules are modified lysosomes that contain at least two distinct classes of cytotoxic effector protein that are expressed selectively in cytotoxic T cells [147].

Such proteins are stored in the lytic granules in an active form, but conditions within the granules prevent them from functioning until after their release. One of these cytotoxic proteins, known as perforin, polymerizes to form transmembrane pores in target cell membranes. Granules that store perforin and granzymes can be seen in armed CD8 cytotoxic effector cells in tissue lesions [147].

Both perforin and granzymes are required for effective cell killing. The granzymes are proteases, so although they have a role in triggering apoptosis in the target cell, they cannot act directly to fragment the DNA. Rather, they must activate an enzyme, or more probably an enzyme cascade, in the target cell. Granzyme B can cleave the ubiquitous cellular enzyme CPP-32, which is believed to have a key role in programmed cell death in all cells. CPP-32 is a caspase and activates a nuclease, called caspase-activated deoxyribonuclease or CAD, by cleaving an inhibitory protein (ICAD) that binds to and inactivates CAD. This enzyme is believed to be the final effector of DNA degradation in apoptosis [147].

Inhibition of granzyme and perforin with monoclonal antibodies significantly de-crease radiation-induced cytotoxicity and survival rate after high doses of radiation. [31] Monoclonal antibodies to granzyme and perforin are considered as a part of the antiradiation antidote therapeutic complex [127].

#### 2.5.5. Microgravity and the Immune System

Immunosuppression during spaceflight has been recognized since the Apollo missions and remains a major health risk for astronauts, particularly in the development of opportunistic viral infections [146]. At the same time, various publications have postulated that microgravity alone could suppress the activity of the human immune system, painting a complex picture of the role of immunity during human spaceflight [155,156,157,158].

A recent investigation in the U.S. Spacelab SLS-1, based on new technology, provided in vitro, in which leukocytes are attached to microcarrier beads, showed that the strong inhibition of activation in microgravity is due to a malfunction of monocytes acting as accessory cells [159]. In fact, interleukin-1 production is nearly nil in resuspended monocytes, whereas T cell activation is doubled in attached cells.

Consistent with prior analyses of NK cell function during long-term spaceflight, data suggest that microgravity (μG) profoundly inhibits CD25 and CD69 expression in NK cell subsets, which are important markers of proliferative and cytotoxic capacity for these cell subsets. Similarly, μG impaired multiple aspects of CD8+ T cell function, including CD25, CD69, and JAK/STAT1 and STAT5 signaling responses. The observed suppression of NK and CD8+ T cell function by μG dovetails with prior documentation of clinically significant impairment of viral pathogen defenses during short and long-term spaceflights, including the re-emergence of latent viruses, such as herpes simplex virus (HSV-1), Epstein-Barr virus (EBV), cytomegalovirus (CMV), and varicella-zoster virus (VZV) [155].

At the molecular level, radiation and/or microgravity are known to negatively impact DNA integrity. To counteract DNA damage, cells have developed specific mechanisms that locate and repair DNA lesions. These mechanisms consist of a network of cellular proteins involved in DNA damage response (DDR) pathways, such as cell cycle regulation, DNA repair, and apoptosis. Generally, cells are able to accommodate moderate DNA damage through these different repair processes; however, space conditions, especially the lack of gravity, may adversely affect the DNA repair process leading to the accumulation of DNA injuries [160,161].

However, the influence of microgravity on the repair of radiation-induced genetic damage in a temperature-conditional repair mutant of the yeast Saccharomyces cerevisiae (rad 54-3) was investigated onboard the IML-1 mission (22th–30th January 1992, STS-42). Cells were irradiated before the flight, incubated under microgravity at the permissive (22 °C) and restrictive (36 °C) temperature, and afterward tested for survival. The results suggest that repair may be reduced under microgravity [160,161].

Pattern recognition receptors not only recognize PAMPs from invading pathogens but also have the ability to sense inflammatory components, also called damage-associated molecular patterns (DAMPs), released from damaged cells. PRRs include TLRs, NOD-like receptors (NLRs), C-type lectin receptors (CLRs), and RIG-I-like receptors (RLRs) [162,163].

## 3. Discussion

**Figure 7:**
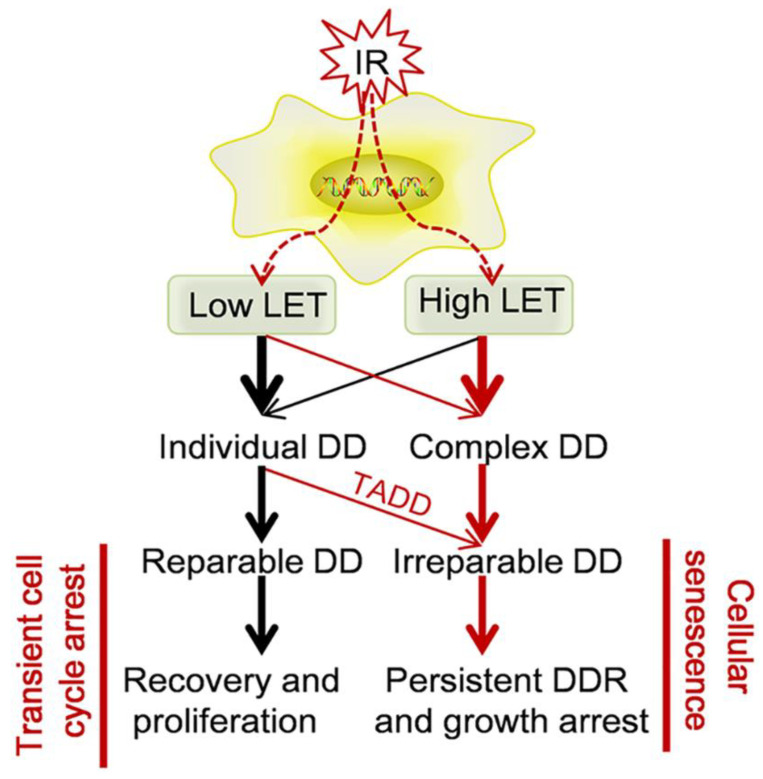
The relationship between energy transfer and the resulting induced cellular and DNA changes. DD is DNA Damage, TADD is Telomere associated DNA Damage, and DDR is DNA Damage Response.

Given the complex nature of the space environment (Figure 7), no single agent or single approach will suffice in our efforts to fully mitigate the health hazards associated with long-duration spaceflight. As such, it is critical that we evaluate a suite of countermeasures that can be used concomitantly and adapt appropriately to the changing dynamics that can result from sporadic SPEs and other periods of high exposure.

The most obvious approach appears to consist of passive countermeasures that are built into the hardware as well as mission parameters. This includes shielding, which currently relies heavily on aluminum and polyethylene, but will have to rely on more effective hydrogen-rich materials and/or new active shielding technologies. Another passive approach relies on transit or exposure time reduction, which could potentially be achieved through a combination of improved propulsion technologies, and mission planning, but will likely have limited impact given the current state of the science.

Research has yielded a promising body of evidence related to the use of nutritional countermeasures in space, documenting (i) Improvements in longevity, mutagenesis, and cognitive impairment on animal models exposed to acute radiation and oxidative stress; (ii) Reduction in anxiety, cognitive, and stress-associated behavioral changes in an animal model; (iii) Safety in humans, and; (iv) Modest efficacy for limited types of oxidative stress-related activities (e.g., exercise-induced muscle fatigue, skin resiliency, anxiety, and stress, etc.) based on animal data, and a small number of human studies.

Generally recognized as safe (GRAS) antioxidant agents such as vitamin C, vitamin E, selenium, N-acetyl cysteine, and alpha-lipoic acid may be recommended as prophylactic agents for deep space missions. Because radiation causes damage at many levels, and because of the inherent differences in radiation types present in space, a combination of agents representing the major classes of prevention molecules may be added to a daily prophylactic regimen. Such a regimen might include one or more of the following agents: polyphenols, terpenes, isoflavones, bioflavonoids, carotenoids, and coenzymes. Several dietary radioprotective agents have been studied for their anti-cataractogenic effects. Tests of agents used to protect against the effects of HZE radiation have been more limited; the few studies conducted have focused on reducing the frequency of mutations in cells, or reducing the number of cancers in exposed animals.

Additionally, since nutritional countermeasures are dietary in nature, and are currently regulated as foods by the Food and Drug Administration, consideration should be paid to NASA’s requirements for food systems. These include safety, stability, palatability, nutrition, resource minimalization, variety, reliability, and usability. While oral supplements inherently satisfy many of these challenges due to their portability, stability, convenience, and bypass of the act of eating/chewing, anyone looking to propose or develop nutritional countermeasures for long-duration spaceflight would be well served to conduct the necessary work to ensure the finished deliverable adheres to these criteria. Moreover, current efforts to develop agricultural means to grow produce in space may ultimately lead to breakthroughs in our ability to supplement the diet with phytonutrients and other fresh food-derived compounds.

Besides their nutritional value and their role in ensuring that caloric requirements are met, dietary agents have the potential to play a significant role in mitigating both early and late health effects of long-duration spaceflight. Radiation-induced damage impacts a wide variety of organs and systems in the human body. It is thus logical that dietary agents such as vitamins, minerals, and antioxidants, known to play a role in a wide variety of metabolic pathways, may play a significant role as first-line prophylactic and potentially mitigating countermeasures. Few compounds offer the type of safety and versatility profile offered by dietary agents, meriting further research.

Beyond the use of nutritional countermeasures, novel and legacy pharmacologic agents currently offer promising solutions ranging from radioprotectors to biological immunomodulators, to radiomitigators and prodromal therapies,

While many diverse compounds can be characterized as radioprotective agents, a broad and practical distinction can be made between radio-modulators, radio-protectors, and radio-mitigators. Radio-modulators are compounds that are administered prophylactically for the purposes of increasing an organism’s baseline resistance to radiation. A classic example is the antioxidant family of compounds, which have been shown to reduce radiation toxicity and carcinogenesis in both cell cultures and animal models [75,101,164]. Researchers have developed combination formulas consisting of multiple radio-modulator agents in an effort to mitigate against multiple pathways of radiation injury while minimizing the side effects associated with the administration of any single agent [165]. A more speculative approach to dietary radioprotection involves the engineering of the gut microbiome via administration of probiotics and prebiotics, undertaken with the aim of promoting or restoring a gut microbiome associated with increased radioresistance. The scientific understanding of the gut microbiome and its role in radiobiology is in its infancy, however, and a more detailed picture of the underlying pathophysiologic processes will have to emerge before any therapeutic avenues can be explored.

Radio-protectors are agents that are administered immediately before an expected radiation insult and protect cellular components directly, often by opposing the action of reactive oxygen species. Radio-protectors are typically pharmaceutical or biological in nature, with few if any, dietary agents meeting this criterion.

Radio-mitigators are a heterogeneous set of compounds that are administered after a radiation insult, for the purposes of accelerating recovery and preventing radiation-related complications. These may range from general supportive measures, such as intravenous fluids, to antibiotics, immune modulators, and certainly nutritional compounds that may play a supportive role in inflammation, electrolyte balance, cellular recovery.

From an immunology perspective, available data suggest that microgravity can result in some degree of immunosuppression. However, current evidence also shows that radiation-induced activation of the immune system via a complementary system plays a significant role in the overall status of immunity, and is a major mechanism of cytotoxicity [160,161,162,163]. The fact that both factors of spaceflight (e.g., radiation exposure and microgravity) induce seemingly opposite effects in human immunity, with one inducing suppression and the other critically activating and possibly over-stimulating it, deserves further research.

**Figure 8:**
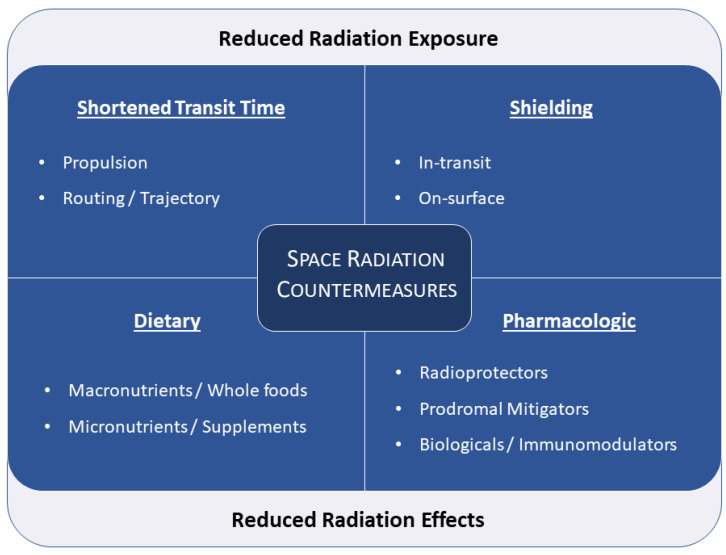
Four types of space radiation countermeasures are evaluated, corresponding to either a “Reduced radiation exposure”, or a “Reduced radiation effects” strategy.

Refer to Figure 8 for a graphic overview of all available countermeasure types reviewed in this article.

The combined influence of microgravity and moderate or high doses of radiation potentially exist as a complicated problem. Although microgravity could change the clinical picture of acute radiation syndromes, it could also increase morbidity and lethality among pilots by drastically activating molecular damage in the cell. Immuno-modulation should be considered as an important therapeutic method for radiation protection. Combined with chemopreventive dietary and pharmaceutical agents, they have demonstrated great results in reducing the biological consequences of radiation and microgravity. Although many details about the influence of microgravity and radiation on human organisms still remain uncertain. Future studies of immune system reactions after different doses and types of radiation combined with microgravity can be very useful for medical support of space missions.

We can summarize the radiation protection strategy for space crew venturing beyond Low Earth Orbit as follows:

1. Provide low weight, neutron-poor shielding in the transit vehicle and surface habitats, to include more heavily shielded storm shelters for the episodic SPE’s that employ easy-to-implement shielding strategies. Surface habitats can utilize naturally shielded areas e.g., lava tubes or regolith if an earth-moving capability is deployed.
2. Have real-time active dosimetry and monitoring (including on EVA suits) which captures high-energy neutrons and includes real-time SPE alarm functions and automated/improved SPE forecasting
3. Develop and fly minimally invasive radiation bioeffects monitoring equipment which may include biomarkers, tissue antioxidant levels, and cytogenetics
4. Validate a radiation countermeasures nutritive and pharmacologic program. This will include an array of field-tested radioprotective molecules and chemoprevention agents, which are shown to be nontoxic, easily administered, of high bioavailability that could be given alone or in combination to ameliorate or prevent radiation cellular and tissue damage. The pharmacokinetics, pharmacodynamics, tissue distribution, safety, and efficacy of such agents need to be thoroughly characterized in both Earth-based simulated conditions and in microgravity with concomitant space radiation.

With the appropriate vehicle and mission design, employing technologically advanced shielding, and a validated radiation countermeasures program, the risk from space radiation should not be prohibitive for the execution of exploration-class missions within our solar system.

## Figures and Tables

**Table 1:** Changes in oxidative stress biomarkers during an example ISS mission [59].

Compound Analyzed.	Example Pre-Flight Value	Example Post-Flight Value	Normal Ranges Observed in-Flight	Maximal Changes Observed Post-Flight (Percentage Change from Pre-Flight)
Total Antioxidant Capacity	1.54	1.47	1.29–1.83	Decreased up to 30%
Superoxide Dismutase (SOD)	1318	1172	1092–1817	Decreased 10–30%
Glutathione Peroxidase	51.5	50.8	27.5–73.6	Decreased 5–15%
Malondialdehyde (MDA)	0.8	0.6	0–2.00	Increased 100–200%
4-OH-alkenal	0.45	0.45	0–2.00	Increased 50–150%
Urinary 8OHdG	3.2	3.7	0.49–7.29	Increased 40–200%

**Table 2:** Natural Sources of Chemical Radioprotective Agents in Plants [75].

Compounds	Sources
N-Acetyl L-Cysteine (NAC), Diallyl sulfide	Onions, garlic, chives, scallions
Sulphoranes, indoles, isothiocyanates	Cruciferous vegetables (e.g., broccoli, kale, cauliflower, cabbage)
Isoflavones and phytoestrogens	Soybeans (e.g., tofu, miso, soy milk)
Ascorbic acid, flavonoids [quercetin, rutin, isoquercetin], terpenes [limonene]	Citrus fruit (e.g., lemon, grapefruit), cherry, oregano, parsley, artichokes
Curcumins	Turmeric
Carotenoids [lutein, lycopene, astaxanthin]	Tomato, carrots, squash, algae, salmon
Polyphenols	Green and black tea, grape, blueberry

**Table 3:** Select components of the GRAS-based Radioprotectant Formula, and associated cellular targets.* [75].

Component	Target
Vitamin B1	Insulin sensitivity, anti-inflammatory
Vitamin B3	Insulin sensitivity, anti-inflammatory
Vitamin B6	Insulin sensitivity, anti-inflammatory, scavenges O_2_^−^
Vitamin B12	Insulin sensitivity, anti-inflammatory
Vitamin E	Antioxidants in the lipid membrane, scavenges O_2_^−^, H_2_O_2_
Astaxanthin	Suppresses NF-κB activation, lipid antioxidant
Bioflavonoids	O_2_^−^, metal chelator
Coenzyme Q10	Mitochondrial support, antioxidant in mitochondria
Green Tea	Antioxidants in the cytosol, scavenges O_2_^−^, H_2_O_2_
Glutathione	Enzymatic antioxidant support, antioxidants in the cytosol
N-Acetylcysteine	Mucolytic, hepatocyte support
Zinc	Neural support (zinc þ antioxidants), insulin sensitivity
Lutein	Retinal radioprotectant, ROS scavenger in the lens
Folic Acid	Antioxidant, maintains glutathione levels, endothelial support
Vitamin C	Antioxidants in the cytosol, scavenges O_2_^−^, H_2_O_2_
Vitamin D	Antioxidants in the lipid membrane
Alpha Lipoic Acid	Mitochondrial support, antioxidant, insulin sensitivity
Chromium	Insulin sensitivity, scavenges H_2_O_2_
Omega-3 FA	Anti-inflammatory, membrane fluidity
Vitamin A	Antioxidant in lipid membrane and retina
Magnesium	Insulin sensitivity, cellular support
Potassium	Insulin sensitivity, cellular support
Selenium	Enzymatic antioxidant support, insulin sensitivity, scavenges H_2_O_2_
Resveratrol	Mitochondrial support, increase SOD activity, scavenges O_2_^−^, H_2_O_2_

* This is not meant to be a complete list or an exhaustive rendering of functions for each of the formula components. The functions are presented only in the context of their innate action. Synergistic and recycling interactions among components are not included.

## Appendix A. Immunomodulatory Background: Impact of Space Radiation Exposure on the Immune System

Radiation induces antigen-specific cellular immunity, with the development of both acute and chronic autoimmune reactions resulting in pro-apoptotic/pro-necrotic intra-cellular signaling pathways [126,145]. For example, it induces expression of immuno-modulatory surface molecules such as Major Histocompatibility Complex (MHC); represents non-self, novel antigens on the cell surface; activates costimulatory molecules; stimulates adhesion molecules; reacts with death receptors, heat shock proteins; and induces mass production secretory molecules (cytokines, inflammatory mediators) in cells, tissues, blood and lymph circulation, stromal, and vascular endothelial cells [95,145,166].

Dendritic cells (DCs) and all antigen-presenting cells (APC) are attracted to this modified molecular microenvironment and undergo maturation and activation upon contact with these modified molecules [146]. After irradiation in vivo, APCs in both categories, professional and non-professional, are activated, and present radiation-induced antigens to the immune system. Radiation-activated professional APCs cooperate with co-stimulatory and pattern-recognition receptors [127,146].

The non-professional APCs express MHC class I molecules, which are also involved in the post-radiational activation of the immune system. Radiation Activated Antigen Presenting Cells (RAAPC) mediate antigen-specific cellular immunity via the presentation of processed antigens to T cells. Radiation-activated T cells then interact with a professional APC, which presents non-self, novel antigens, recognized by their T cell receptor. Following radiation exposure in vivo, Helper T cells recognize exogenous antigen presented on MHC class II; whereas cytotoxic T cells can recognize endogenous antigen presented on MHC class I.

Most cells in the body can present antigen to CD8+ cytotoxic T cells via MHC class I; however, the term “antigen-presenting cell” is often used specifically to describe professional APCs. Such cells express MHC class I and MHC class II molecules and can stimulate CD4+ helper T cells as well as cytotoxic T cells [95,130,145,147].

APCs can also present foreign and self-lipids to T cells and NK cells by using the CD1 family of proteins, which are structurally similar to the MHC class I family [167]. APCs and DCs have phagocytosed modified molecules from irradiated, apoptotic, necrotic cells, and they usually migrate to the vast network of lymph vessels, where they are then carried away to the lymph nodes. Redistribution of lymphocytes and migration from blood circulation to lymphatic circulation can explain the decrease in lymphocyte counts and DC volume in blood circulation, in the early hours following irradiation. APCs usually interact with T cells inside of lymph nodes.

After migration to lymphatic circulation, APCs and DCs change the surface expression of MHC and co-stimulatory molecules, as well as increase the production of cytokines. The internalized non-self, novel antigens are digested into smaller peptides containing epitopes, which are then presented to T cells by the MHC [168,169,170]. B cells reside in the lymph node. Once their receptors bind to an antigen, they can interact with activated helper T cells [168]. A DC that interacts with an already-activated helper T cell can become licensed [170,171,172].

Licensing occurs through the interaction of co-stimulatory molecules including B7 and CD40 on the dendritic cell, with CD28 and CD40 ligand on the T cell. Only licensed dendritic cells are able to activate cytotoxic T cells. T cell licensing of DCs is key for activation of cytotoxic T cells for many non-self, autoimmune antigens [171,172]. In MHC class I and class II molecules, only certain epitopes of an internalized peptide can be presented. These epitopes could be specific for radiation and are termed immunodominant epitopes or specific determinants of radiation.

APCs naturally have a role in fighting tumors, via stimulation of B and cytotoxic T cells to respectively produce antibodies against tumor-related antigens and to kill malignant cells [169,170]. However, it appears that irradiated cells stimulate the activation of the immune system quite similar to the activation of the immune system against cancer cells. Dendritic cells, presenting radiation-specific antigens to T cells, are key to the process of immune modulation [169,170,171,172].

Immune-therapies of acute radiation syndromes have included treating the patients with two important stages of immunomodulation therapy:

1. Reduce activation of acute immunogenic, autoimmune reactions, and prevent mass destruction of irradiated cells by immune cells and active proteins, such as MAC, and granzyme.
2. Gradually increase numbers of dendritic cells or radiation-specific T cells for slow elimination of irradiated cells.

However, new therapies have turned to genetically engineered, artificial APCs designed to prime the immune system to attack non-self-antigens belonging to irradiated cells. Some artificial APCs are derived from human cells; others are acellular, containing MHC proteins, co-stimulatory molecules, and the necessary peptides. Those therapies could be effective at the second stage of immunomodulation [169,170,171,172].

Further enhancement of radiation-mediated inflammation and cell death can be achieved via the administration of a group of biological molecules that mimic radiation toxicity and can induce similar to radiation, biological consequences. Radiation-mediated immune modulation currently remains unquantified and poorly understood. A major research effort will be required to elucidate mechanisms of action.

With a thorough understanding of this phenomenon, we believe that ionizing radiation could be optimized for use with antiradiation vaccines. Antiradiation antidote generated antigen-specific cellular immunity, with a high level of radioresistance [126,127]. Ionizing radiation (IR) induces specific changes in biological molecules, causes DNA lesions, and leads to a wide range of cytotoxic reactions and cytotoxic bio-logical polymers [132,140,173,174,175].

IR can induce molecular alterations in irradiated cells, making them an effective target for several types of cytotoxicity and immune cell-mediated pathologic apoptosis and/or necrosis [126,173]. The effect of IR on antigen presentation by MHC class I molecules was studied on models of tumor cells and irradiated cells. [139,140]. The ability of IR to influence the immune response against irradiated cells and tissues became a target for the development of novel immunotherapies (IT) of acute radiation syndrome. The group of immunomodulatory agents became considerably important for practical and clinical use [126,127,132].

Radiation induces a type of cell death in a subset of susceptible irradiated cells or tissues, which can then modulate the peptide repertoire, change antigenicity from self- to non-self; activate antigen uptake by APC, and presentation APCs with activation of acute immune reactions [139,140]. Radiation-induced novel antigens or radiation antigens activate a cascade of cytotoxic T lymphocyte recognition of irradiated cells [139,140]. Novel proteins were made in response to gamma-irradiation, resulting in new peptides presented by MHC class I molecules, which were recognized by cytotoxic T cells [139,140].

We show in our previously demonstrated results that IT and immune-prophylaxis of acute radiation syndrome are successful in eradicating severe forms of radiation toxicity and significantly decrease morbidity and mortality from high doses of radiation [126,127,128,132].

Radiation induces several types of cytotoxicity:

- Radiation-induced Antibody-Dependent Cellular Cytotoxicity (RADCC).
- Radiation-induced Complement-Dependent Cytotoxicity (RCDC).
- Radiation-Occurring Antibody-Independent Cellular Cytotoxic (ROAICC) activity of circulating leucocytes toxicity.
- ATM-Controlled Radiation Toxicity (ATMCRT)

Reactive Oxygen Species are the important contributors to radiation cytotoxicity, DNA damage, significant macromolecule damage. The radiation damage can lead to cell pathology, altered cell function, and apoptosis or cell necrosis. [ROSCC]

### Appendix A.1. Radiation-Induced Antibody-Dependent Cellular Cytotoxicity (RADCC)

Radiation-induced zntibody-dependent cellular cytotoxicity (RADCC), also referred to as radiation antibody-dependent cell-mediated cytotoxicity, is a mechanism of cell-mediated immune defense whereby an effector cell of the immune system actively lyses irradiated cell, whose membrane-surface antigens demonstrated non-self, antigenicity, and have been bound by specific antibodies [139].

### Appendix A.2. Radiation-Induced Complement-Dependent Cytotoxicity (RCDC)

Radiation-induced complement-dependent cytotoxicity (RCDC) is an effector function of IgG and IgM cytotoxic antibodies. After cytotoxic antibodies are bound to surface antigen on irradiated cells, the classical complement pathway is triggered by bonding protein C1q to these antibodies, resulting in the formation of a membrane attack complex (MAC) and target cell lysis. This process of cytotoxicity considering as specific for irradiated tumor cells, which express non-self, antigens on the cell surface. However, it could be a similar process for just irradiated cells [132,176,177,178].

### Appendix A.3. Radiation-Occurring Antibody-Independent Cellular Cytotoxic Activity (ROAICC) of Circulating Leukocytes

Exposure to total-body radiation induces hematological changes. Decreases in blood cell count after acute radiation doses of γ-ray or proton radiation exposure, at the doses and dose-rates expected during a solar particle event (SPE), are reported in the ferret model system [174,176,177,178]. Following the exposure to γ-ray or proton radiation, the ferret peripheral total white blood cell (WBC), and lymphocyte counts decreased whereas neutrophil count increased within 3 h [176,178].

At 48 h after irradiation, the WBC, neutrophil, and lymphocyte counts decreased in a dose-dependent manner but were not significantly affected by the radiation type (γ-rays versus protons) or dose rate (0.5 Gy/minute versus 0.5 Gy/hour) [176,178]. The loss of these blood cells could accompany and contribute to the physiological symptoms of acute radiation syndrome (ARS) [140,176,177]. However, some scientific results demonstrated total white cell counts and lymphocytes counts decreased in blood circulation and rising in the lymphatic system and lymphatic circulation.

### Appendix A.4. TM Controlled Radiation Toxicity (ATMCRT)

Ataxia-telangiectasia is such an interesting disease, which demonstrated gene mutation and the dependence of radiosensitivity from the single gene mutation. Ataxia-telangiectasia is due to mutations of the ATM gene, located on chromosome 11q22-23 [133,134,135,136,137,138,139,179,180].

It is a good model for searching novel methods of radiation, detection, radiation protection, and cancer therapy. Serine/threonine-protein kinase that activates checkpoint signaling upon double-strand breaks (DSBs), apoptosis, and genotoxic stresses such as ionizing ultraviolet A light (UVA), thereby acting as a DNA damage sensor [133,134,135,136,137,138,139,141,163,179]. A mutation of the ATM gene is responsible for aberrant repairing of the breaks of double-strand DNA. Because of this defect, cell response to different pathogenic triggers, such as ionizing radiation and alkylating agents, is impaired. As a consequence, cells death occurs in susceptible tissues such as the cerebellum, and malignant proliferation arises [133,134,135,136,137,138,139,179,180]. However, activation of normal Serine/threonine-protein kinase can significantly increase radioresistance.

Methods of immune-modulation, as a part of therapeutic countermeasures based on repairing damaged DNA after irradiation, play an important role in the development of radioresistance, which greatly supports astronauts’/cosmonauts’ benefit for protection against radiation [173,179,180].
